# California Healthy Places Index and COVID-19 hospitalization risk: a patient-level analysis

**DOI:** 10.1186/s12889-026-27732-3

**Published:** 2026-05-14

**Authors:** Dian Gu, Janice Y. Tsoh

**Affiliations:** 1 The Center for Tobacco Control Research and Education, University of California, San Francisco, USA; 2 Division of General Internal Medicine, San Francisco General Hospital, University of California, San Francisco, USA; 3 Department of Psychiatry and Behavioral Sciences, School of Medicine, University of California, San Francisco, USA; 4 University of California Helen Diller Family Comprehensive Cancer Center, San Francisco, USA; 5 Asian American Research Center on Health, University of California, San Francisco, USA

**Keywords:** Patient-level analysis, Health inequity, Social determinants of health, Neighborhood social determinants, Patient-level COVID-19 hospitalization, California Healthy Place Index

## Abstract

**Background:**

Research has shown that inequalities in COVID-19 outcomes are influenced by individual-level characteristics and specific measures of neighborhood characteristics. Understanding the association between composite neighborhood-level social determinants of health, particularly newly developed determinant indices, and COVID-19 outcomes is important. The objective of this study is to contribute to the literature by evaluating the association between the California Healthy Places Index (HPI)—an extensive yet relatively underutilized measure of neighborhood health and well-being—and a significant health outcome at the patient level, hospitalization related to COVID-19.

**Methods:**

Utilizing a retrospective cohort study design, we examined electronic health record data from adult patients who tested positive for COVID-19 between 2/1/2020 and 2/3/2022 at an academic medical center in Northern California. The primary outcome was hospitalization within 14 days of the first COVID-19 diagnosis. We used both unadjusted and multivariable logistic generalized linear mixed models with random intercepts at the county and ZIP code (nested within county) levels to examine the relationship between California HPI quartiles—where the lowest quartile represents the most disadvantaged neighborhoods—and COVID-19 hospitalization. The multivariable model adjusted for individual-level characteristics as covariates.

**Results:**

Of the 21,831 patients included in the cohort, 1,355 (6.2%) were hospitalized with COVID-19. The results demonstrated a dose–response relationship between the California Healthy Places Index (HPI) and patient-level COVID-19 hospitalization in the unadjusted model, and this association remained significant after adjusting for covariates. In the multivariable model, patients living in neighborhoods within the lowest HPI quartile had the highest odds of hospitalization (adjusted odds ratio [AOR] = 1.90, 95% confidence interval [CI] = 1.28–2.83; average marginal effect [AME] = 0.04, 95% CI = 0.01–0.06) compared to those in the highest quartile, followed by patients in the second and third quartiles.

**Conclusions:**

Study findings indicate that among patients with COVID-19, residence in more disadvantaged neighborhoods was associated with higher hospitalization rates. These results could encourage investigation into neighborhood-level social determinants of health, such as those measured by the California HPI, and their associations with health inequities. They may also inform future research exploring causal mechanisms underlying these inequities and could guide evaluation of targeted interventions to address them.

**Supplementary Information:**

The online version contains supplementary material available at 10.1186/s12889-026-27732-3.

## Introduction

Health inequities were amplified during the COVID-19 pandemic in the US [1]. For example, African American and Asian American patients were more likely to have admission to intensive care unit due to COVID-19 than White patients [2]. Educational and occupational disadvantages were important factors for COVID-19 mortality among working-age adults [3]. All these findings reveal that inequalities in COVID-19 outcomes exist due to individual-level characteristics.

Aside from individual-level disparities, neighborhood characteristics also contribute to COVID-19 outcome inequities at the macro level. Neighborhood characteristics encompass various social and environmental factors that influence a wide range of health outcomes [4], and are considered a key domain of social determinants of health [4]. Several studies have explored neighborhood indices and their association with COVID-19 outcomes, such as mortality [5–12]. For example, limited resources available to rural hospitals and areas have been linked to higher COVID-19 mortality rates compared to urban regions [5]. Furthermore, lower neighborhood income has been identified as a positive predictor for invasive mechanical ventilation and ICU admission among individuals hospitalized with COVID-19 [6]. Neighborhood disadvantage, as measured by the Area Deprivation Index(ADI), has been shown to predict in-hospital COVID-19 mortality [11]. Similarly, U.S. counties with high social vulnerability, as measured by the Social Vulnerability Index(SVI), are associated with an increased risk of COVID-19-related mortality [8]. These findings underscore the impactful role of neighborhood-level social determinants in driving health inequities during the pandemic.

The current literature has primarily focused on specific measures of neighborhood characteristics, capturing only certain aspects of neighborhood conditions, or on widely used national composite indices for general population, including ADI and SVI [5–11, 13, 14]. However, there has been limited research on newer composite neighborhood indices developed in recent years that incorporate additional relevant parameters for public health [9, 12]. One such index is the California Healthy Places Index (HPI) [15], representing a new generation of neighborhood composite indices. Unlike ADI and SVI, which primarily focus on socioeconomic deprivation [16], the California HPI incorporates additional environmental and infrastructural factors. These include environmental conditions, such as air and water pollution levels, and aspects of social cohesion and community engagement, such as voting participation. By integrating these diverse factors, the California HPI offers a more comprehensive indicator of neighborhood health and well-being, making it a potentially valuable tool for public health research, policy decisions and strategic planning in California [15]. Compared with prior studies using ADI or SVI [8–11, 13, 14], less is known about whether the broader, California-specific HPI, which incorporates additional environmental and infrastructural dimensions of neighborhood context, is associated with patient-level COVID-19 hospitalization. To augment the literature, this study examined the association between the California HPI, and COVID-19 hospitalization within a university health system in California. California HPI incorporates an array of indicators spanning 8 domains- economics, education, health care access, housing, neighborhood conditions, pollution/clean environment, social, and transportation, into a single neighborhood-level indexed HPI Score [15]. It offers a comprehensive overview of health and well-being in each neighborhood [15]. Developed by the Public Health Alliance of Southern California in 2018 [15], it has been adopted throughout California and put into different actions to fairly allocate funds with the goal of improving the neighborhood health [15]. California Department of Public Health has utilized California HPI to conduct public health surveillance of COVID-19, and support equitable COVID-19 vaccine distribution [17, 18], but the associations with COVID-19 severe outcomes, such as hospitalization, were still understudied using statistical modelling. Only one study found that Black veterans with COVID-19 living in lower California HPI neighborhood had higher risk of COVID-19 hospitalization after accounting for individual and neighborhood race/ethnicity segregation confounders [9].

The objective of this study is to empirically apply the California HPI, a composite index that captures multiple facets of neighborhood context, to examine its association with patient-level COVID-19 hospitalization while accounting for patients’ characteristics. Given the observational design of the study, we aim to identify associations rather than establish causal relationships. The research question is: How is the California HPI associated with hospitalization risk among individuals with COVID-19? We hypothesize that individuals living in disadvantaged neighborhoods, as measured by the California HPI, will show an association with a higher risk of hospitalization within 14 days of a COVID-19 diagnosis compared to those living in more advantaged neighborhoods. Findings may help inform policymakers about patterns in neighborhood-level correlates of health outcomes and guide the prioritization of public resources and targeted interventions to improve COVID-19–related health equity. Although COVID-19 is no longer classified as a pandemic, it remains a public health threat, highlighting the need for continued efforts to prevent severe outcomes [19]. This study can also have implications beyond COVID-19 sequalae by adding evidence regarding the scope of the application of HPI-based approach in health inequity studies, which could prepare the medical and public health investigators to allocate investments efficiently to help curtail future large-scale outbreaks of infectious diseases.

## Methods

### Study design and data source

This study is part of the COVID Electronic Health Record (EHR) Cohort at the University of Wisconsin (CEC-UW) (ClinicalTrials.gov: NCT04506528), a retrospective cohort study supported by the National Cancer Institute. Twenty-one healthcare systems in the US participated in this study and provided selected EHR from their COVID-19 patients. Participating health systems extracted selected structured EHR data elements, including patient sociodemographic and clinical characteristics, clinical encounter data, pre- and post-COVID ICD-10 diagnoses, and laboratory test results, and transmitted them to the coordinating center for harmonization [20]. Free-text clinical notes were not used in this study. The University of California, San Francisco (UCSF) Health System is one of the participating health systems and the only one with language information available in the EHR extract. While language is not the primary focus of our study, UCSF Health’s diverse patient population includes many immigrants and non-English speakers, enabling analyses that adjust for language and consider cultural and linguistic diversity alongside neighborhood conditions. This supports a more comprehensive examination of the HPI in the context of health equity. We extracted EHR data from the UCSF Health System for the period between February 1, 2020, and February 3, 2022. The study sample focused on adult patients, aged 18 or older, residing in California, who had a valid California ZIP code for obtaining the California HPI. The study sample included patients residing in 54 counties, constituting 93% of the California’s 58 counties. The date of each patient’s first confirmed positive COVID-19 PCR test within the UCSF Health System was designated as the index date.

### Outcome variable

The outcome, defined as COVID-19 hospitalization by two criteria, either (1) hospitalized within 14 days of the index date (+/- 7 days centered at the admission date) and/or having an ICD-10 COVID-19 diagnosis (U07.1 or J12.82) during the hospitalization and within 90 days of the index date [21] and (2) hospitalized for COVID-19 for at least 24 h, or mortality within 24 h of admission, or transfer to the intensive care unit within 24 h of admission.

### Independent variables

**Fig. 1:**
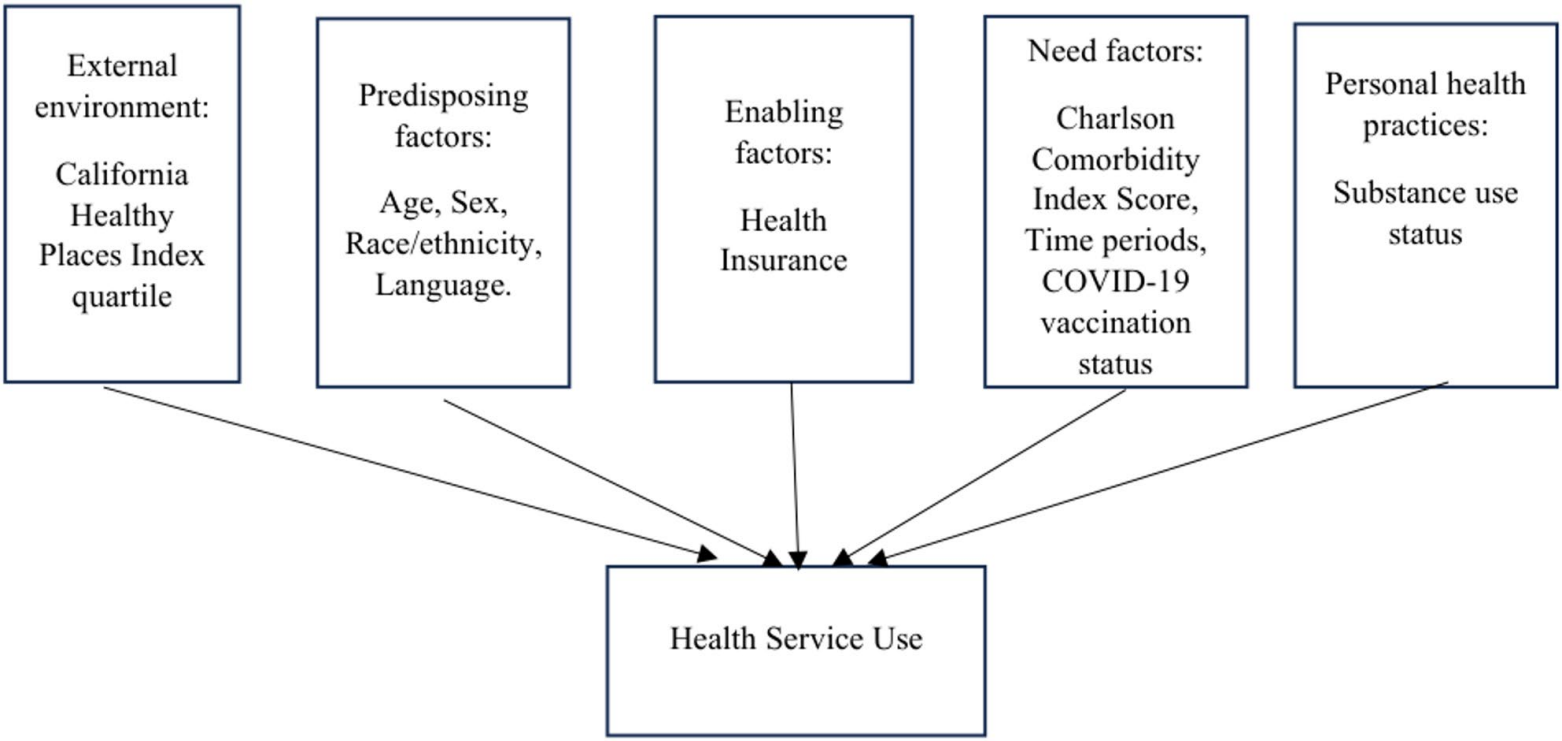
The expanded Andersen Behavioral Model applied in the study

All the independent variables were selected based on the expanded Andersen Behavioral Model to explain the use of health services [22], taking into consideration the data elements available in the EHR data. As depicted in Fig. 1, the model is composed of five main constructs: (1) predisposing factors; (2) enabling factors; (3) need factors; (4) personal health practices; and (5) external environment. This study focuses on the California HPI and its association with patient-level COVID-19 hospitalization with consideration of other individual characteristics from the main constructs of the Andersen Behavioral Model, which are described below.

### Key independent variable

The key independent variable is the California Healthy Places Index (HPI) quartile, categorized using 0.25, 0.5, and 0.75 cutoff thresholds. It belongs to the external environment construct of the expanded Andersen Behavioral Model and is derived from California’s HPI Version 3.0 score, which was developed based on a social determinants of health framework. California HPI 3.0 is the latest version of HPI and assigns a percentile-based score (0–1) to each area (e.g., census tract, zip code) in California based on neighborhood-level conditions associated with health [15]. Areas with lower HPI scores have fewer opportunities for residents to lead healthy lives. Each area’s HPI score is derived from 23 indicators in 8 socioeconomic domains [15]. We used the patients’ ZIP codes on the index date to link to the ZIP code-level HPI 3.0 score file. ZIP-code-level HPI estimates have been used in prior California COVID-19 research, including to characterize inequities in mortality and to inform equity-focused vaccination outreach [23, 24].

### Other independent variables

Other independent variables were individual-level characteristics measured on the index date, and belong to the other four constructs of the expanded Andersen Behavioral Model (Fig. 1). Predisposing factors were age, sex, race/ethnicity, and preferred language. Race/ethnicity serves as a proxy for individual-level social determinants of health and reflects systemic inequities. Enabling factors, conceptualized as factors influencing health care access, were measured by health insurance. Need factors were assessed by 3 variables: the Charlson Comorbidity Index(CCI) score [25] as a proxy of health comorbidity burden —calculated as a weighted score based on diagnoses within one year of the index date—along with the time periods during the pandemic and vaccination status (full, partial, not vaccinated). We grouped the time periods in reference to vaccines availability: (1) index date on or before 12/31/2020 when vaccines were not accessible to patients; (2) between 1/1 and 3/31/2021 when vaccines were available to selected groups of individuals such as individual patients at high-risk; and (3) 4/1/2021 or after when vaccines became available to all adult patients. We defined full vaccination as two weeks after the second dose in a two-dose primary series, such as the Pfizer or Moderna vaccines, or two weeks after a single-dose vaccine, such as the Johnson & Johnson vaccine. Partial vaccination referred to the status between not vaccinated and fully vaccinated. Lastly, personal health practices included substance use, defined as any use of tobacco, cannabis, or alcohol. Predisposing and enabling variables, vaccination status, and substance use were obtained from structured fields in the EHR. The CCI was calculated from ICD-10 based diagnosis data in the EHR [26].

### Study sample

**Fig. 2:**
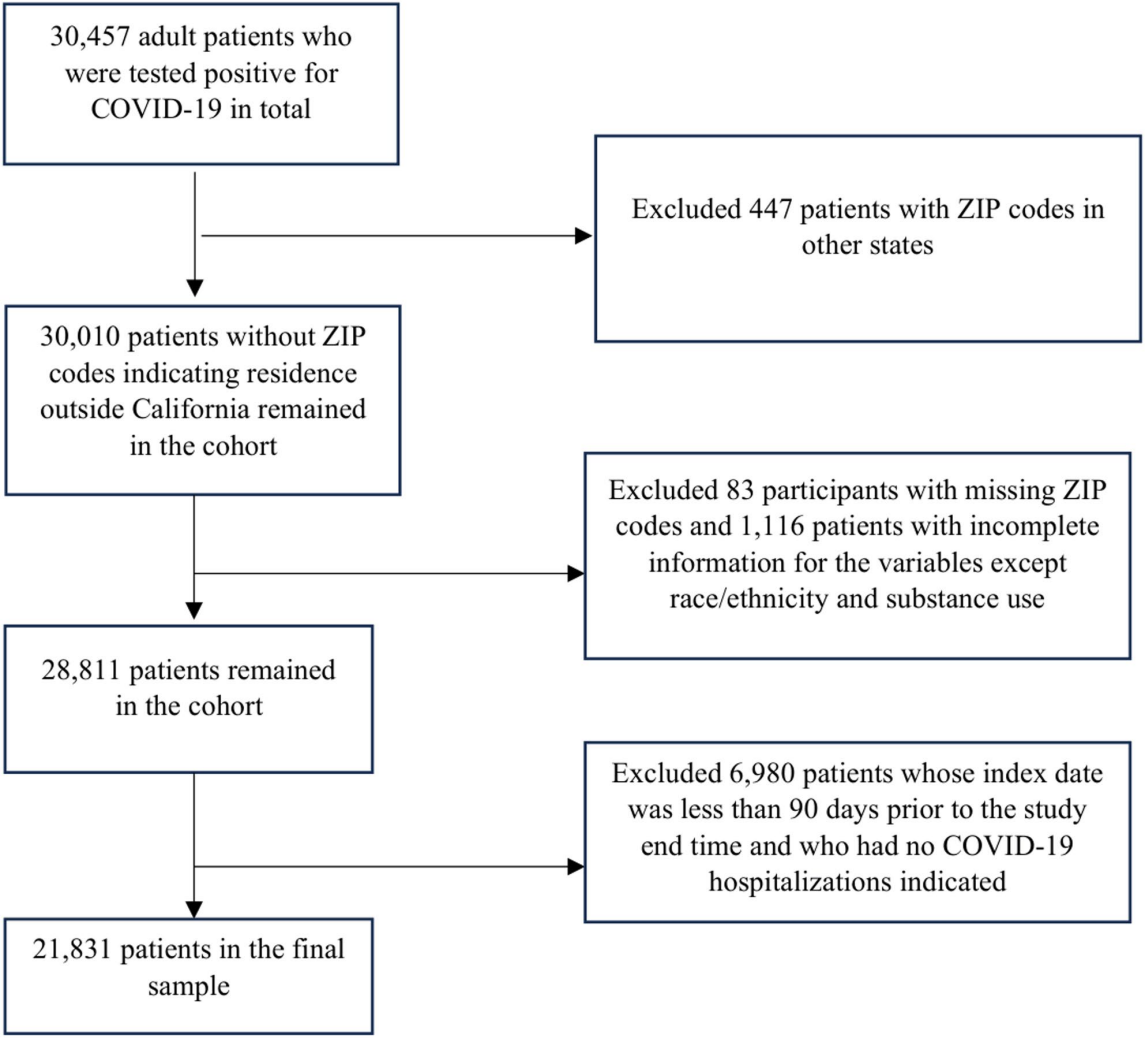
Selection of the final study sample

The study sample included 30,457 adult patients who tested positive for COVID-19. We first excluded 447 patients with ZIP codes from other states, yielding a cohort of 30,010 patients. In addition, we excluded 83 patients with missing ZIP codes. Next, we excluded 1,116 patients with incomplete data for independent variables, except for race/ethnicity and substance use status. In total, 1,199 patients (4.0%) were removed due to missing ZIP codes or incomplete data. We excluded patients with ZIP codes from other states to maintain geographic homogeneity in our study sample. According to the statistical guidance [27, 28], removing such a small proportion of cases is inconsequential to model estimation. Among the 28,811 patients remained, more than 10% had missing data for race/ethnicity or substance use status, so we created an “unknown” category for them. To ensure all the patients can be traced back to 90 days prior to the end of the study for the COVID-19 hospitalization outcome, patients whose index date was less than 90 days prior to the study end time and had no COVID-19 hospitalization indicated were excluded from the analyses. A total of 6,980 were excluded due to the index date criterion; the final study sample contained 21,831 adult COVID-19 patients (Fig. 2).

### Statistical analyses

We first computed descriptive statistics to summarize all the independent variables. Then, we used χ² tests to determine if there were significant differences in the proportion of patients with COVID-19 hospitalization across the subgroups of the independent variables. Lastly, to examine the association between the California HPI and COVID-19 hospitalization, we used logistic generalized linear mixed models with two levels of random intercepts (county and ZIP code nested within county), estimated using Laplace approximation and robust standard errors. Model convergence was confirmed, and the estimated random-effects variance components were examined to support the appropriateness of the nested random-intercept specification. These models accounted for clustering at both the ZIP code (neighborhood) and county (regional) levels. We conducted both an unadjusted model and an adjusted model that controlled for all other independent variables. All analyses were performed using SAS V.9.4 (SAS Institute, Cary, North Carolina, USA). Significance was defined by *P* ≤ 0.05.

## Results

Table 1 demonstrated the sample characteristics. Of the final study sample, 1,416 (6.5%) and 14,327 (65.6%) patients lived in neighborhoods in the lowest and highest California HPI quartiles, respectively. This distribution reflects the characteristics of patients receiving care within the UCSF Health system and its referral catchment, rather than the population distribution of the broader study region. While it does not necessarily indicate sampling bias within the health system, it may differ from the underlying regional population. There were about equal number of patients in each age group and more female (56.3%) than male. More patients were non-Hispanic White (34.8%) and the majority of patients were English Speakers (89.4%), followed by Spanish (6.0%) and Chinese speakers (1.1%). Also, more patients were covered by public insurance (43.2%), had 0 CCI score (80.2%), having index date on or before 12/31/2020 (54.7%), were not vaccinated at index date (81.8%) and did not use any substance (44.2%). Among the 21,831 patients, 1,355(6.2%) were hospitalized with COVID-19 (Table 1). The χ² tests revealed significant differences in proportion of hospitalized patients with respect to all the independent variables. Supplemental Table 1 presented the *P*-values for pairwise post-hoc χ² tests.

**Table 1:** Sample characteristics and proportion of patients with COVID-19 hospitalization by selected characteristics

	Entire Sample *N* (%)	COVID-19 Hospitalization		
		Yes *N* (%)	No *N* (%)	*P*-value
Entire sample	21,831(100%)	1,355(6.2%)	20,476(93.8%)	
External Environment				
California Healthy Places Index quartile				< 0.0001
Lowest quartile	1,416 (6.5%)	152 (10.7%)	1,264 (89.3%)	
Second quartile	2,876 (13.2%)	280 (9.7%)	2,596 (90.3%)	
Third quartile	3,212 (14.7%)	208 (6.5%)	3,004 (93.5%)	
Highest quartile	14,327 (65.6%)	715 (5.0%)	13,612 (95.0%)	
Predisposing factors				
Age				< 0.0001
18–34	5,934 (27.2%)	250 (4.2%)	5,684 (95.8%)	
35–49	5,277 (24.2%)	242(4.6%)	5,035 (95.4%)	
50–64	5,459 (25.0%)	336 (6.2%)	5,123 (93.8%)	
65+	5,161 (23.6%)	527 (10.2%)	4,634 (89.8%)	
Sex				< 0.0001
Female	12,292 (56.3%)	656 (5.3%)	11,636 (94.7%)	
Male	9,539 (43.7%)	699 (7.3%)	8,840 (92.7%)	
Race/Ethnicity				<0.0001
Non-Hispanic White	7,591 (34.8%)	394 (5.2%)	7,197 (94.8%)	
Hispanic	4,481 (20.5%)	369 (8.2%)	4,112 (91.8%)	
Non-Hispanic Black	1,764 (8.1%)	176 (10.0%)	1,588 (90.0%)	
Non-Hispanic Asian	3,141 (14.4%)	266 (8.5%)	2,875 (91.5%)	
Non-Hispanic Other	1,640 (7.5%)	90 (5.5%)	1,550 (94.5%)	
Unknown	3,214 (14.7%)	60 (1.9%)	3,154 (98.1%)	
Language				< 0.0001
English	19,527 (89.4%)	991 (5.1%)	18,536 (94.9%)	
Spanish	1,302 (6.0%)	178 (13.7%)	1,124 (86.3%)	
Chinese	239 (1.1%)	70 (29.3%)	169 (70.7%)	
Other	763 (3.5%)	116 (15.2%)	647 (84.8%)	
Enabling factors				
Health insurance				<0.0001
Commercial	7,252 (33.2%)	269 (3.7%)	6,983 (96.3%)	
Public	9,432 (43.2%)	1,027 (10.9%)	8,405 (89.1%)	
Other	5,147 (23.6%)	59 (1.1%)	5,088 (98.9%)	
Need factors				
Charlson Comorbidity Index score				< 0.0001
0	17,498 (80.2%)	794 (4.5%)	16,704 (95.5%)	
1	1,454 (6.7%)	143 (9.8%)	1,311 (90.2%)	
≥ 2	2,879 (13.2%)	418 (14.5%)	2,461 (85.5%)	
Time periods				<0.0001
On or before 12/31 2020	11,951 (54.7%)	556 (4.7%)	11,395 (95.3%)	
1/1–3/31/2021	4,214 (19.3%)	234 (5.6%)	3,980 (94.4%)	
4/1/2021 or after	5,666 (26.0%)	565 (10.0%)	5,101 (90.0%)	
Vaccination status at index date				<0.0001
Not vaccinated	17,850 (81.8%)	1,075 (6.0%)	16,775 (94.0%)	
Partial	600 (2.7%)	22 (3.7%)	578 (96.3%)	
Full	3,381 (15.5%)	258 (7.6%)	3,123 (92.4%)	
Personal health practices				
Substance use status				< 0.0001
Yes	4,795(22.0%)	525(11.0%)	4,270(89.0%)	
No	9,665(44.2%)	704(7.3%)	8,961(92.7%)	
Unknown	7,371(33.8%)	126(1.7%)	7,245(98.3%)	

**Fig. 3:**
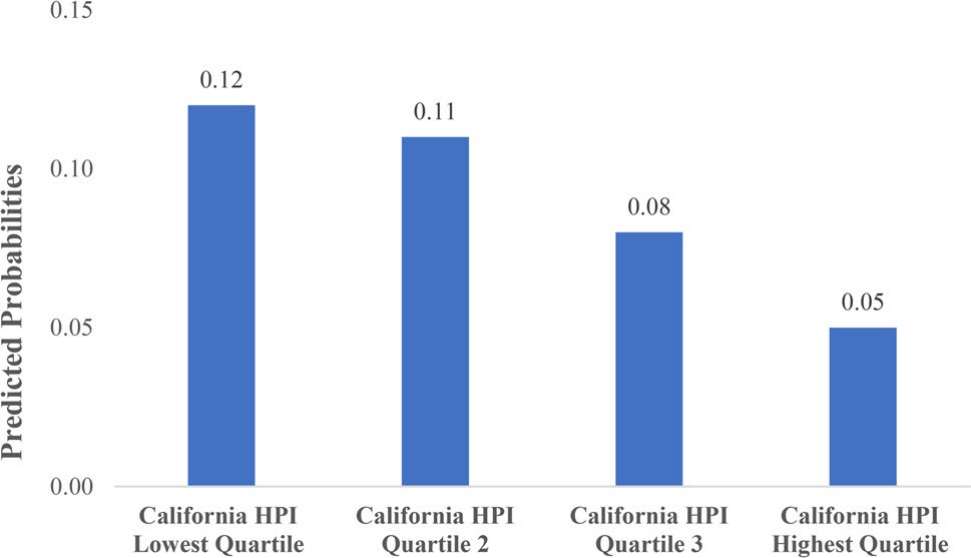
Predicted Probabilities of COVID-19 Hospitalization by California HPI Quartiles in Model 1 (Unadjusted Model). Note: HPI=Healthy Places Index

**Fig. 4:**
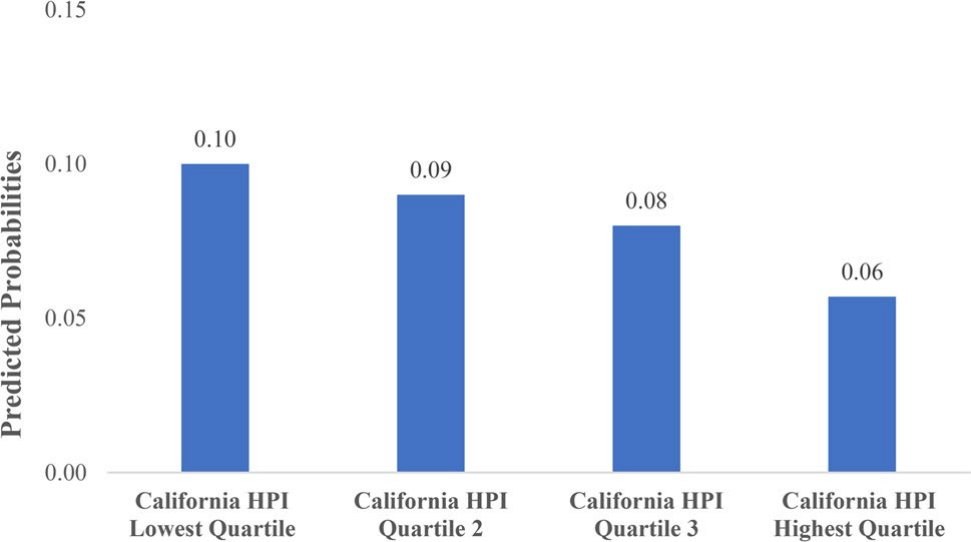
Predicted Probabilities of COVID-19 Hospitalization by California HPI Quartiles in Model 2 (Adjusted Model). Note: HPI=Healthy Places Index. This multivariable model adjusted for predisposing (age, sex, race/ethnicity, language), enabling (health insurance), need factors (Charlson Comorbidity Index score, time periods, COVID-19 vaccination status), and personal health practices factors (substance use status)

Tables 2 and 3 presented the results from the unadjusted and multivariable logistic generalized linear mixed models, and Figs. 3 and 4 illustrated the predicted probabilities of COVID-19 hospitalization across California HPI quartiles. The unadjusted model showed a dose-response relationship between lower HPI quartiles and higher odds of hospitalization. Patients residing in neighborhoods within the lowest HPI quartile had the highest odds of hospitalization (OR = 2.75, 95% CI 1.82–4.16; AME = 0.07, 95% CI 0.05–0.10) compared to those in the most advantaged quartile. This was followed by patients in the second HPI quartile (OR = 2.39, 95% CI 1.59–3.59; AME = 0.06, 95% CI 0.04–0.08) and those in the third quartile (OR = 1.78, 95% CI 1.38–2.30; AME = 0.03, 95% CI 0.02–0.05). After adjusting for individual-level characteristics, the dose-response pattern remained, though the strength of the associations was attenuated. Patients in the lowest HPI quartile continued to have the highest odds of hospitalization (AOR = 1.90, 95% CI 1.28–2.83; AME = 0.04; 95% CI 0.01–0.06), followed by those in the second quartile (AOR = 1.82, 95% CI 1.28–2.58; AME = 0.03; 95% CI 0.01–0.05) and third quartile (AOR = 1.53, 95% CI 1.16–1.98; AME = 0.02; 95% CI 0.00–0.03). In terms of individual-level characteristics (Supplemental Table 2), the characteristics associated with higher odds of hospitalization included older age, being male, being racial and ethnic minorities, speaking languages other than English, covered by public insurance, having higher CCI scores, being not vaccinated and using substance.

**Table 2:** Unadjusted logistic generalized linear mixed model of COVID-19 hospitalization

	OR (95% CI)	*P*-value	AME (95% CI)
External environment			
California Healthy Places Index quartile			
Lowest quartile	2.75(1.82–4.16)	< 0.0001	0.07(0.05–0.10)
Second quartile	2.39(1.59–3.59)	< 0.0001	0.06(0.04–0.08)
Third quartile	1.78(1.38–2.30)	< 0.0001	0.03(0.02–0.05)
Highest quartile	REF		

*OR* Odds ratio, *AME* Average marginal effect, *95% CI* 95% Confidence Interval

**Table 3:** Multivariable logistic generalized linear mixed model of COVID-19 hospitalization

	AOR (95% CI)	*P*-value	AME (95% CI)
External environment			
California Healthy Places Index quartile			
Lowest quartile	1.90(1.28–2.83)	0.0015	0.04(0.01–0.06)
Second quartile	1.82(1.28–2.58)	0.0008	0.03(0.01–0.05)
Third quartile	1.53(1.16–1.98)	0.0021	0.02(0.00–0.03)
Highest quartile	REF		

*AOR* Adjusted odds ratio, *AME* Average marginal effect, *95% CI* 95% Confidence Interval

This multivariable model adjusted for predisposing, enabling, need factors, and personal health practices factors

## Discussion

Consistent with our hypothesis, this study found a dose-response relationship between California HPI and patient-level COVID-19 hospitalization based on both the unadjusted and multivariable model. The patients living in neighborhoods with lowest HPI quartile had the highest odds of hospitalization, followed by those living in the second and third HPI quartile. This association between lower HPI and greater odds of COVID-19 hospitalization is consistent with the prior study focusing on the veterans [9], reinforcing the utility of this indices in assessing COVID-19 outcome risks. Beyond COVID-19 research, it aligns with a study suggesting a dose-response relationship between the California HPI and all-cause mortality in California [29]. All these findings shed light on the need for future research among different vulnerable populations and the examination of other COVID-19 outcomes to confirm this finding and further delineate the scope of application of the California HPI.

### Recommendations

The California HPI was previously utilized in a practical application when the state introduced a vaccine equity metric based on the HPI [18]. This strategy involved allocating additional vaccine doses to the hardest-hit quarter of the state, as identified by the HPI, to facilitate equitable vaccine distribution. This approach supported efforts to reopen the state safely and sustainably. To strengthen the call for concrete public action based on our findings using ZIP-code–level California HPI, future research could apply spatial analytic approaches, such as choropleth mapping to visualize spatial patterns and spatial regression analysis that accounts for spatial dependence, at more granular geographic levels (e.g., census tract–level measures). These approaches could improve geographic precision, better capture localized neighborhood variation, and refine the understanding of neighborhood effects. Such analyses could provide new insights into localized inequities and help identify geographic areas where targeted outreach or interventions could be prioritized and evaluated.

This study contributes to the literature examining links between composite neighborhood-level social determinants of health index and COVID-19 outcomes [8–12]. Compared to some other composite indices (e.g., ADI, SVI), this HPI is a relatively new tool and warrants further evaluation of its advantages and disadvantages. A study [30] comparing the California HPI, ADI, and SVI in assessing social risk within California found that HPI and ADI are less correlated than HPI and SVI. Moreover, while ADI primarily focuses on socioeconomic factors, SVI covers a broader range of domains by including additional demographic variables. In contrast, HPI provides a more comprehensive assessment of communities’ relative health within California. However, HPI does not currently include a component of racial and ethnic segregation, a form of structural racism. Although the previous study concluded that it can adequately captures risk specifically for COVID-19 hospitalization [9], further investigation is necessary to determine whether the absence of certain factors affects the HPI’s performance in capturing COVID-19 outcomes and other health risks. This is important because accurately assessing the impact of social determinants on health outcomes requires composite measures to comprehensively reflect the multifaceted nature of neighborhoods. Direct comparative analyses of HPI versus other neighborhood indices (e.g., ADI, SVI) were beyond the scope of this study and should be examined in future research.

### Public health implications

After the declaration of the end of COVID-19 as a public health emergency, COVID-19 has not disappeared but become endemic. Our study findings can have important implications beyond the pandemic. On an individual and health system scale, integrating neighborhood indices such as the California HPI into electronic health records as a risk modifier could enhance risk stratification and support more proactive, context-informed care for patients living in high-risk areas. For example, HPI-linked data could be used to identify patients who may benefit from early clinical monitoring (e.g., proactive symptom check-ins), targeted outreach (e.g., follow-up contact after COVID-19 diagnosis), or enhanced care coordination (e.g., linkage to neighborhood-based services). Integrating neighborhood-level information such as the HPI or similar indices into clinical workflows may help guide health systems in tailoring interventions to patients’ contextual risk. Nonetheless, further research is needed to determine how best to operationalize these approaches and evaluate their impact on clinical outcomes. On a population and policy scale, our findings could support prioritizing neighborhoods in the lowest HPI quartiles for targeted interventions. Public health agencies could allocate resources to these neighborhoods through strategies such as targeted outreach by health liaisons and improved access to preventive and acute healthcare services. Such context- or place-informed approaches could guide preparedness and response strategies for future disease outbreaks and other public health emergencies. It is important to note that these implications are derived from observed associations and do not establish causality; thus, further research should evaluate whether HPI-informed strategies improve population health outcomes.

### Limitations

Findings should be interpreted with caution, as several methodological and sampling limitations may affect the internal validity of the results and their generalization to other settings and populations. In terms of limitations affecting internal validity of the findings, we note the following. First, the EHR data lacks information on certain individual-level characteristics that might affect the outcome, such as immigration status and length of residence in the U.S. for patients who prefer languages other than English. Future well-designed research is needed to reassess the associations found in the study. Second, our HPI measure is based on ZIP codes rather than census tracts, which typically have less within-unit variation [31]. ZIP code was the most granular geographic identifier consistently available in the EHR; census tract was not available for linkage. Because ZIP codes are larger geographic units and can contain heterogeneous neighborhood conditions, ZIP-based linkage may introduce area-level exposure misclassification and may dilute neighborhood gradients and attenuate observed associations [32, 33]. Previous research suggests that indices based on census tracts, such as ADI, more effectively capture localized health disparities and provide greater precision for place-based interventions than those based on ZIP codes [34, 35]. Future research should explore the advantages and limitations of using HPI at the census tract level versus the ZIP code level. Additionally, future studies could incorporate more granular geographic units, such as census tract–level measures to better capture localized neighborhood variation and refine the understanding of neighborhood effects. Third, the high rate of missingness in race/ethnicity and substance use status in our EHR system may be related to informative presence bias [36]. Thus, missingness may not be random, and we did not use multiple imputation; multiple imputation could introduce additional bias under missing-not-at-random conditions [37]. We created “unknown” categories, which is a pragmatic approach commonly used in EHR-based research [38–40]. However, this strategy may not fully resolve missing-data bias, as “unknown” categories could be highly heterogeneous, encompassing individuals with different underlying values and different reasons for missingness, and the approach may bias association estimates in observational studies because confounding may be only partially adjusted among individuals with missing covariate data [41, 42]. As a result, the observed associations may be either underestimated or overestimated, and the direction of bias cannot be determined with certainty because the “unknown” group is likely heterogeneous and the missingness mechanism is uncertain [41, 42]. Targeted interventions for patients, clinicians and support staff are needed to enhance data collection across patient subgroups. Fourth, the timing and method of COVID-19 diagnosis—such as access to testing or variation in clinical presentation severity—may differ across neighborhoods due to structural barriers, differences in healthcare access, or employment constraints. But our data does not include any COVID-19 onset information. To account for pre-existing health conditions that could contribute to the risk of COVID-19 diagnosis or severity, we included the Charlson Comorbidity Index in the model. Furthermore, the California HPI includes domains such as transportation and healthcare access, which capture neighborhood-level differences in proximity to care. Although this partially accounts for disparities in diagnosis timing and access, it may not fully reflect barriers such as job-related constraints, which can also influence when and how individuals seek care and receive a diagnosis. As a result, some of the observed disparities in hospitalization may reflect differences in diagnostic pathways. Nevertheless, our findings offer practical utility for health services planning by highlighting system-level and structural factors relevant to resource allocation.

Regarding external validity or generalizability limitations, we note the following. First, because the HPI is state-specific, the findings may not be generalizable to other regions. However, the results can still guide the development of similar neighborhood indices in other states. Second, the study focused solely on one severe COVID-19 outcome– hospitalization. Continued research on other severe outcomes (e.g., long-COVID) is needed. Third, our study includes only patients from a single health system. As a major tertiary/quaternary referral center, UCSF Health serves patients requiring specialized services and higher-acuity care, including referrals or transfers. Patients seeking care at UCSF may differ from those receiving care elsewhere in terms of health-seeking behavior. These selection processes may shape the cohort’s composition, limiting its external generalizability to the broader population. Accordingly, it reduces the ability to draw broad public health conclusions without further contextualization. However, our sample included patients from 54 of California’s 58 counties (93%), offering substantial geographic and demographic diversity. Future research should validate our findings in diverse healthcare settings to enhance generalizability.

## Conclusion

This study found that the California HPI can be a valuable tool for identifying neighborhoods vulnerable to adverse COVID-19 health outcomes. When combined with individual characteristics, it could inform tailored intervention efforts to improve COVID-19 outcomes and likely shape broader public health strategies beyond COVID-19. This underscores the importance of incorporating these factors into policy-making. As composite indices evolve, future research on health inequity should monitor and examine the California HPI and other newly developed indices that incorporate neighborhood-level social determinants of health, such as the Childhood Opportunity Index for children [43], and the Structural Racism Effect Index for the general population [44]. Finally, these findings should be validated across other healthcare systems and geographic settings before informing large-scale policy decisions or broader public health strategies.

## Supplementary Information

Supplementary Material 1.

## Data Availability

The datasets analyzed during the current study are not publicly available, but are available from the corresponding author upon reasonable request.

## Abbreviations

HPI: Healthy Places Index
AOR: Adjusted Odds Ratio
CI: Confidence Interval
