# Supplementary material for: California Healthy Places Index and COVID-19 hospitalization risk: a patient-level analysis
Source: BMC Public Health. 2026 May 14;26:2068. doi: 10.1186/s12889-026-27732-3 (PMC13340339; doi:10.1186/s12889-026-27732-3)
Supplement: Supplementary file 1 — Supplementary Material 1. [file 12889_2026_27732_MOESM1_ESM.docx]

**Supplemental Tables**

| **Supplemental Table 1: Subgroup P-values for Differences in COVID-19 Hospitalization Across Categories of Non-Binary Independent Variables** | |
| --- | --- |
|  | P-value |
| **External Environment** |  |
| California Healthy Places Index quartile |  |
| Lowest vs. Second quartile | <.0001 |
| Lowest vs. Third quartile | <.0001 |
| Lowest vs. Highest quartile | <.0001 |
| Second vs. Third quartile | 0.0003 |
| Second vs. Highest quartile | <.0001 |
| Third vs. Highest quartile | 0.1580 |
| **Predisposing factors** |  |
| Age |  |
| 18-34 vs. 35-49 | 0.3360 |
| 18-34 vs. 50-64 | <.0001 |
| 18-34 vs. 65+ | <.0001 |
| 35-49 vs. 50-64 | 0.0003 |
| 35-49 vs. 65+ | <.0001 |
| 50-64 vs. 65+ | <.0001 |
| Race/Ethnicity |  |
| Non-Hispanic White vs. Hispanic | <.0001 |
| Non-Hispanic White vs. Non-Hispanic Black | <.0001 |
| Non-Hispanic White vs. Non-Hispanic Asian | <.0001 |
| Non-Hispanic White vs. Non-Hispanic Other | 0.6241 |
| Non-Hispanic White vs. Unknown | <.0001 |
| Hispanic vs. Non-Hispanic Black | 0.0280 |
| Hispanic vs. Non-Hispanic Asian | 0.7161 |
| Hispanic vs. Non-Hispanic Other | 0.0003 |
| Hispanic vs. Unknown | <.0001 |
| Non-Hispanic Black vs. Non-Hispanic Asian | 0.0766 |
| Non-Hispanic Black vs. Non-Hispanic Other | <.0001 |
| Non-Hispanic Black vs. Unknown | <.0001 |
| Non-Hispanic Asian vs. Non-Hispanic Other | 0.0002 |
| Non-Hispanic Asian vs. Unknown | <.0001 |
| Non-Hispanic Other vs. Unknown | <.0001 |
| Language |  |
| English vs. Spanish | <.0001 |
| English vs. Chinese | <.0001 |
| English vs. Other | <.0001 |
| Spanish vs. Chinese | <.0001 |
| Spanish vs. Other | 0.3363 |
| Chinese vs. Other | <.0001 |
| **Enabling factors** |  |
| Health insurance |  |
| Commercial vs. Public | <.0001 |
| Commercial vs. Other | <.0001 |
| Public vs. Other | <.0001 |
| **Need factors** |  |
| Charlson Comorbidity Index score |  |
| 0 vs. 1 | <.0001 |
| 0 vs. ≥2 | <.0001 |
| 1 vs. ≥2 | <.0001 |
| Time periods |  |
| On or before 12/31 2020 vs. 1/1-3/31/2021 | 0.0197 |
| On or before 12/31 2020 vs. 4/1/2021 or after | <.0001 |
| 1/1 - 3/31/2021 vs. 4/1/2021 or after | <.0001 |
| **Personal health practices** |  |
| Vaccination status at index date |  |
| Not vaccinated vs. Partial | 0.0164 |
| Not vaccinated vs. Full | 0.0004 |
| Partial vs. Full | 0.0005 |
| Substance use status |  |
| Yes vs. No | <.0001 |
| Yes vs. Unknown | <.0001 |
| No vs. Unknown | <.0001 |

| **Supplemental Table 2: Multivariable logistic generalized linear mixed model of COVID-19 hospitalization for predisposing, enabling, need factors, and personal health practices factors** | | | |
| --- | --- | --- | --- |
|  | AOR (95% CI) | P-Value | AME (95% CI) |
| **Predisposing factors** | | |  |
| Age |  |  |  |
| 35-49 | 0.99(0.72–1.36) | 0.9342 | 0.00(-0.01-0.01) |
| 50-64 | 1.13(0.69-1.84) | 0.6319 | 0.01(-0.01-0.02) |
| 65+ | 1.51(1.25-1.82) | <.0001 | 0.02(0.01-0.04) |
| 18-34 | REF |  |  |
| Sex |  |  |  |
| Male | 1.38(1.18-1.62) | <.0001 | 0.02(0.01-0.03) |
| Female | REF |  |  |
| Race/Ethnicity |  |  |  |
| Hispanic | 1.33(1.08-1.64) | 0.0069 | 0.02(0.00-0.03) |
| Non-Hispanic Black | 1.82(1.55-2.12) | <.0001 | 0.04(0.02-0.05) |
| Non-Hispanic Asian | 1.49(1.21-1.84) | 0.0002 | 0.02(0.01-0.04) |
| Non-Hispanic Other | 1.25(1.00-1.54) | 0.0459 | 0.01(-0.01-0.03) |
| Unknown | 0.72(0.56-0.94) | 0.0148 | -0.01(-0.03-0.00) |
| Non-Hispanic White | REF |  |  |
| Language |  |  |  |
| Spanish | 2.56(2.12-3.09) | <.0001 | 0.06(0.04-0.09) |
| Chinese | 3.74(3.04-4.58) | <.0001 | 0.10(0.06-0.15) |
| Other | 3.13(2.17-4.54) | <.0001 | 0.09(0.06-0.11) |
| English | REF |  |  |
| **Enabling factors** | | |  |
| Health Insurance |  |  |  |
| Public | 1.93(1.66-2.25) | <.0001 | 0.04(0.03-0.05) |
| Other | 0.52(0.42-0.65) | <.0001 | -0.02(-0.03--0.01) |
| Commercial | REF |  |  |
| **Need factors** | | |  |
| Charlson Comorbidity Index Score |  |  |  |
| 1 | 1.25(1.07-1.45) | 0.0051 | 0.01(0.00-0.02) |
| ≥2 | 1.61(1.33-1.94) | <.0001 | 0.03(0.02-0.04) |
| 0 | REF |  |  |
| Time periods |  |  |  |
| On or before 12/31 2020 | 0.35(0.24-0.51) | <.0001 | -0.07(-0.08--0.05) |
| 1/1 - 3/31/2021 | 0.37(0.32-0.43) | <.0001 | -0.07(-0.08--0.05) |
| 4/1/2021 or after | REF |  |  |
| Vaccination status |  |  |  |
| No | 1.70(1.26-2.31) | 0.0006 | 0.03(0.02-0.04) |
| Partial | 0.62(0.46-0.84) | 0.0023 | -0.02(-0.03-0.00) |
| Full | REF |  |  |
| **Personal health practices** | | |  |
| Substance use status |  |  |  |
| Yes | 1.33(1.25-1.41) | <.0001 | 0.02(0.01-0.03) |
| Unknown | 0.33(0.23-0.46) | <.0001 | -0.05(-0.06--0.04) |
| No | REF |  |  |
